# Health-related quality of life in parents of adolescents one year into the COVID-19 pandemic: a two-year longitudinal study

**DOI:** 10.1186/s12955-022-02069-8

**Published:** 2022-12-01

**Authors:** Gudrun Rohde, Sølvi Helseth, Siv Skarstein, Milada Småstuen, Hilde E. T. Mikkelsen, Kristin Haraldstad

**Affiliations:** 1 University of Agder, Kristiansand, Norway; 2 Department of Health and Nursing, Faculty of Health and Sport Sciences, University of Agder, Kristiansand, Postbox 422, 4604 Norway; 3 Department of Nursing and Health Promotion, Faculty of Health Sciences, Oslo Metropolitan University, Oslo, Norway

**Keywords:** Parents of adolescents, HRQOL, Pain, Loneliness, Resilience, Longitudinal

## Abstract

**Aim:**

For many adults, their role as a parent is a vital part of their life that may influence their health-related quality of life (HRQOL) and vary with the age of their child. The aim of the present study was to describe and compare sociodemographic and psychological factors, pain and HRQOL in parents of adolescents assessed at baseline and 2 years later,—during the COVID-19 pandemic.

**Methods:**

A longitudinal study of 309 parents from the general Norwegian population was conducted. The parents were chosen based on their adolescent’s school belonging and responded to a web-based questionnaire. We used data collected at baseline (T1), when the adolescents were aged 14–15 years (2018/2019), and two years later (T2), in 2021, when the COVID-19 pandemic was ongoing. The response rate was 55%. HRQOL was assessed using RAND-36. Data were analysed using McNemar tests, paired samples t-tests and multiple linear regression analyses.

**Results:**

Of the participants, 82% were mothers and 18% fathers. From T1 to T2, the average pain score increased, 1.6 (95% CI [-1,4; 1.8]) vs 1.8 (95% CI [1,6; 2.0]), the pain interference emotion score increased, 1.6 (95% CI [1.3; 1.9]) vs 1.8 (95% CI [1.5; 2.1]), and a larger proportion reported pain duration > 3 months (44% vs 50%, *p* = 0.014). The parents were more lonely, 12.8 (95% CI [12.3; 13.3]) vs 13.7 (95% CI [13.2; 14.2]), and reported lower RAND-36 mental component summary (MCS) scores, 52.2 (95% CI [51.3; 53.2]) vs 50.9 (95% CI [49.8; 52.0]). There were no significant associations between gender, sociodemographic factors, psychological factors, pain at T1 and changes in RAND-36 physical component summary (PCS). A positive change in MCS from T1 to T2 was predicted by working part time, B = 5.22 (95% CI [1.05; 9.38]) (ref no paid work) and older age, B = 0.24, (95%CI [-001; 0.42]), and there was a negative change with stress, B = -17.39, (95%CI [-27.42; -7.51]).

**Conclusion:**

The parents experienced more pain and were lonelier, and more reported reduced mental HRQOL. However, the changes appear to be of limited clinical significance.

**Supplementary Information:**

The online version contains supplementary material available at 10.1186/s12955-022-02069-8.

## Introduction

Quality of life is increasingly used as goal in political programs, research settings, including clinical practice, population health surveys and in the general adult population. In a health promotion context, health-related quality of life (HRQOL) is defined as a multidimensional construct that includes an individual’s subjective perspective on the physical, psychological, social and functional aspects of health [1]. In previous studies of the general adult population, demographic characteristics such as male gender, higher educational level [2, 3], belonging to a higher socio-economic class [4], having a high income, being married or cohabiting and being employed [5] have been associated with high HRQOL. By contrast, having pain [6], older age [7], long-term disease or health problems [1] and an unhealthy lifestyle [4] are associated with low HRQOL. Longitudinal HRQOL data from the general population are scarce.

For many adults, their role as a parent is an important part of their life. Being a parent of adolescent may be challenging, and the role of a parent may influence HRQOL and vary with the age of their child. Adolescence is characterised by significant physical, cognitive and psychosocial changes that are related to self-identity, peer relationships, development of autonomy and sexuality [8]. Parent-adolescent’s conflicts may increase as the adolescent’s needs for autonomy and independence increase, and they may show some resistance to family rules and roles. Furthermore, in many Western cultures, the passage to adulthood regarding parent-adolescent relationships is viewed as functional, helping adolescents to individualize from their parents, try more things on their own, and develop their own competence[9, 10]. For most adolescents, their relationships to the parents improve as they move into the later adolescent years. However, more serious conflicts between youth and their parents during the early adolescent years may result in more serious challenges[10][11], which can influence parents’ HRQOL. For example, parents can experience pain and high levels of stress, potentially affecting their HRQOL and ability as caregivers [12, 13]. In parents of young children in the general Swedish population, mental health problems have predicted low parental HRQOL. Furthermore, families with at least one individual experiencing problems and in need of assistance rated their HRQOL as lower than families without such problems [14].

The global public health crisis caused by the COVID-19 pandemic has negatively affected the health and well-being of individuals and societies. In the general population, the early stages of the pandemic were often associated with increased levels of economic and social impact, stress and depressive and anxiety symptoms [15–18]. However, longitudinal studies during that time also revealed signs of resilience and the ability to adapt [15]. In countries like Norway the COVID-19 pandemic has been handled rather well, including governmental actions to secure workplaces, and giving some economic compensations. Despite of this, some Norwegian inhabitants experienced stress and insecurity following the consequences of the pandemic [19, 20].

Longitudinal studies on how parents’ HRQOL might change while their children are adolescents seem to be scarce, and previous studies have mainly focused on parents of adolescents with special conditions or diseases [12, 21–23]. Studying parents’ psychosocial and physical well-being also in the general population is important, since adolescents often need support from their parents, and especially during an ongoing crisis [12, 24, 25]. Based on findings of associates and predictors of HRQOL in adults from the general population, and the theoretical basis such as the revised conceptual model of HRQOL by Wilson and Cleary [26] indicating relationships between symptoms, functioning, characteristics of the individual and the environment and HRQOL, the aim of the present study is to describe and compare sociodemographic and psychological factors, pain and HRQOL in parents of adolescents assessed at baseline and 2 years later, during the COVID-19 pandemic.

### Sample and data collection

The present study is part of the ‘*Start Young* Quality of Life and Pain in Generations’ project and elaborates on the findings of our previous study of the HRQOL of parents of adolescents [27]. The ‘Start Young – Quality of Life and Pain in Generations’ [14] is a Norwegian longitudinal study of adolescents and their parents. The present study used data collected from the parents at baseline (T1), when the adolescents were aged 14–15 years (November 2018 to April 2019) [14], and data collected from January to February 2021 (T2), when the adolescents were aged 16–17 years and the COVID-19 pandemic was ongoing.

At T1 one parent of an adolescent aged 14–15 years from 22 schools (that varied in size and localization) were invited to participate in the study. Potential participants in the study were 1,663 adolescent–parent dyads from the participating schools. In total, 696 adolescents (41.8%) and 561 parents (33.7%) filled in the questionnaire at T1[27]. All parents who participated at T1 (*N* = 561) were sent a text message at T2 with a link to the survey and an invitation to take part. They received up to three reminders if they did not complete the second survey. In total, 309 parents (response rate: 55%) completed the survey at both time points and were included in the analysis of this study. According to approval from the Norwegian Centre for Research Data, this was the only way we were allowed to approach the parents.

The data were collected using a web-based questionnaire at both time points. We used a safe data server to store the collected data [37]. The questionnaires from T1 were linked to the questionnaires at T2 through their ID number. All study procedures were approved by the Norwegian Centre for Research Data (Ref: 60,981).

All questionnaires had previously been translated into Norwegian and validated. All questionnaires that used sum scales showed satisfactory Cronbach’s alpha values reported in a previous publication [27]. Most questions included a neutral option, resulting in all items being answered. For the parents we collected data only concerning them, and not of their child.

### Instruments

The instruments and questionnaires for data collection at T1 and T2 were the same.

#### Demographic variables

The first part of the questionnaire included self-reported data on demographic variables such as gender, date of birth, marital status, education, household income, absence from work and geographical region. Possible region-related differences were included in multiple analyses because a previous study showed a negative pattern within some psychosocial variables in one of the regions, such as more people being on disability benefits and the use of anti-depressive medication [28].

#### Pain, HRQOL, self-efficacy, self-esteem, loneliness and stress

*Pain* was measured using the Brief Pain Inventory (BPI) [29] and some questions from the Lübeck Pain-Screening Questionnaire (LPQ) [30]. The BPI measures pain occurrence, worst pain severity, pain inference and number of pain locations, and it has well-established validity and reliability internationally and in Norway [29, 31]. Pain interference questions were completed by those who scored ≥ 1 on the ‘pain on average’ question (indicating that they had pain) [31]. Respondents who rated ≥ 1 on this question of the BPI were given two follow-up questions from the LPQ about pain duration and pain frequency. The LPQ is a structured self-report questionnaire that is used to estimate the prevalence and consequences of pain [30]. The Norwegian LPQ has satisfactory feasibility, content and face validity [32]. Two questions derived from the Norwegian ‘Pain, youth and self-medication study’ (SUS) [33] were used to measure the intake of over-the-counter (OTC) analgesics. The respondents were first asked about OTC analgesic intake during the previous 4 weeks; those who answered ‘yes’ were asked about the frequency of intake.

The RAND-36 was used to assess *HRQOL.* RAND-36 is a generic questionnaire that includes eight domains: general health, bodily pain, physical function, role limitations (physical), mental health, vitality, social function and role limitations (emotional). These domains can be combined into physical and mental sum scales that reflect physical (physical component summary [PCS]) and mental (mental component summary [MCS]) health. The RAND-36 scales were scored according to published scoring procedures, and each scale was transformed to range from 0 to 100, with 100 representing excellent health [34–37]. Delta scores for RAND-36 PCS and MCS were calculated by subtracting mean T1 scores from mean T2 scores. At baseline the Cronbach’s α values for the current study were 0.89 for mental health, 0.89 for bodily pain, 0.83 for general health, 0.87 for social function, 0.89 for physical function, 0.93 for role limitation physical, and 0.87 for role limitation emotional.

*Self-efficacy* was measured using a general self-efficacy (GSE) scale consisting of 10 items [38, 39]. The scale was designed to measure a general sense of perceived self-efficacy and aims to predict the ability to cope with daily demands and adaptation after a stressful experience. The instrument has a four-point scale from 1 (completely wrong) to 4 (completely right), and scores on each item are summed and divided by 10 to form a GSE score ranging from 1–4. Higher scores indicate higher GSE levels. The questionnaire has been shown to be reliable and valid [27, 39]. Cronbach’s α in this study was 0.89.

*Self-esteem* was measured using a short version of the Rosenberg Self-Esteem Scale (RSES) [40], for which respondents rated four statements on self-perception on a 4-point Likert scale ranging from 1 (strongly disagree) to 4 (strongly agree). Higher values would indicate higher levels of self-esteem. The respondents’ scores on each item were summed up and divided by 4 to create an RSES score of 1–4. The questionnaire has been shown to be reliable and valid [27][41]. Cronbach’s α in this study was 0.73.

*Loneliness* was measured using the eight-item version of the revised UCLA Loneliness Scale (ULS-8) [42]. This instrument is a short version of the widely used 20-item revised ULS-20 [43]. ULS-8 uses a 4-point Likert scale with values ranging from ‘never’ to ‘always’. The total score ranges from 8 to 32 points, and higher scores suggest a higher degree of loneliness [43]. The ULS-8 questionnaire was translated into Norwegian and validated as part of the Start Young study [27, 44]. Cronbach’s α in this study was 0.87.

*Stress* was measured using the Perceived Stress Questionnaire (PSQ) [45–47], which is a 30-item questionnaire referring to the previous 4 weeks and answered using a 4-point rating scale ranging from 1 (almost never) to 4 (almost always). The answers were recoded so that higher values indicated higher levels of perceived stress. The resulting PSQ total score was linearly transformed to a number between 0 and 1 using the equation PSQ = (raw value – 30) / 90 [45]. The Norwegian version of the instrument has been shown to have good reliability and validity [27, 47]. Cronbach’s α in this study was 0.87.

### Data analysis

All statistical analyses were conducted using IBM SPSS Statistics (version 27). Descriptive statistics were calculated for all variables, and data have been presented as means with standard deviations (SDs) or as counts and percentages for categorical variables, as appropriate. Paired-samples *t* tests were used to compare differences in continuous data scores between T1 and T2, for all the parents and both the genders separately, while McNemar tests were used to compare pairs of categorical data. To compare baseline characteristics between responders to non-responders at T2, we used Chi-square for categorical variables and independent t-test for the continuous ones.

Multiple linear regression analysis (ENTER procedure in the SPSS) was used to examine the impact of baseline characteristics on changes in HRQOL (delta RAND-36 PCS and MCS) from T1 to T2. Independent variables in the multiple regression analyses were the demographic variables of age, gender, marital status (cohabiting/living alone), region, education, employment status, absence from work, household income, self-efficacy, self-esteem, pain, loneliness and stress. These variables were significantly associated with HRQOL at T1 and also identified in previous studies [12]. The regression analyses were adjusted for RAND-36 PCS and MCS scores at baseline. The potential multicollinearity between the independent variables were evaluated and were considered satisfactory. The level of significance was set at 0.05. All the analyses were considered exploratory, so no correction for multiple testing was done, and *p*-values < 0.05 were considered exploratory. All tests were two-sided. All analyses were performed using SPSS version 17.

## Results

The socio-demographic and pain characteristics of the participants are provided in Tables 1 and 2.

**Table 1:** Characteristics of the sample (*N* = 309) at baseline (2019) and 2 years follow-up (2021), including 252 women and 57 men with valid responses at both times

Demographic	All *N* = 309		*p*-value	Mothers *N* = 252 (82%)		*p*-value	Fathers *N* = 57 (18%)		*p*-value
	T1 (2019)	T2 (2021)		T1 (2019)	T2 (2021)		T1 (2019)	T2 (2021)	
Age, years mean (SD)	45.6 (4.6)			45.3 (4.5)			47.1 (4.8)		
*Living situation*									
Married/cohabitating	249 (81%)	244 (79%)	0.351	204 (81%)	201 (80%)	0.532	45 (79%)	42 (75%)	0.223
Single	17 (5%)	17 (5%)		16 (6%)	16 (6%)		2 (3%)	1 (2%)	
Divorced or separated	41 (13%)	45 (15%)		32 (12.7%)	33 (13%)		9 (16%)	12 (21%)	
Widowed	2 (1%)	3 (1%)		1 (0.4%)	2 (1%)		1 (2%)	1 (2%)	
*Education*			0.443			0.611			n.a
Compulsory education	0	0							
Post-compulsory 1–3 years	6 (2%)	8 (3%)		5 (2%)	8 (3%)		1 (1.5%)		
Post-compulsory 3 years	22 (7%)	22 (7%)		21 (8%)	19 (8%)			3 (5%)	
Certificate of apprenticeship	30 (10%)	30 (10%)		26 (10%)	25 (10%)		4 (7%)	5 (9%)	
University < 4 years	75 (24%)	69 (22%)		57 (23%)	55 (22%)		18 (32%)	14 (25%)	
University ≥ 4 years	176 (57%)	180 (58%)		143 (57%)	145 (57%)		33 (58%)	36 (61%)	
*Employment status*			0.137			0.163			0.311
Full-time	233 (75%)	241 (78%)		181 (72%)	186 (74%)		52 (91%)	55 (96%)	
Part-time	52 (17%)	41 (13%)		49 (19%)	40 (16%)		3 (5%)	1 (2%)	
Not working	24 (8%)	27 (9%)		22 (9%)	26 (10%)		2 (4%)	1 (2%)	
*Absence from work in last 3 months*			0.087			0.178			n.a
None	203 (66%)	231 (75%)		162 (65%)	180 (71%)		41 (72%)	51 (89%)	
1–4 days	69 (22%)	40 (13%)		58 (23%)	35 (14%)		11 (19%)	5 (9%)	
5–7 days	9 (3%)	10 (3%)		6 (2%)	10 (4%)		3 (5%)	0	
8–10 days	3 (1%)	3 (1%)		3 (1%)	3 (1%)		2 (4%)	1 (2%)	
More than 10 days	26 (8%)	25 (8%)		23 (9%)	24 (10%)		0	1	
*Household income (NOK)*			0.167			0.215			0.675
< 250,000	1	2 (1%)		1	2 (1%)		0	0	
250,000–450,000	20 (7%)	17 (5%)		18 (7%)	16 (6%)		2 (4%)	1 (2%)	
451,000–750,000	52 (17%)	48 816%)		45 818%)	41 (16%)		7 (12%)	7 (12%)	
751,000–1,000,000	72 (23%)	60 (19%)		68 (27%)	57 (23%)		4 (7%)	3 (5%)	
> 1,000,000	164 (53%)	182 (59%9		120 (48%)	136 (54%)		44 (77%)	46 (81%)	

Categorical data are presented as number (%) and continuous variables as mean (SD). McNemar tests were used to compare differences in categorical variables and paired-sample *t* tests for continuous data

**Table 2:** Description of pain and differences between T1 and T2 for all (*N* = 309) and for woman (*N* = 252) and men (*N* = 57) separately

	All *N* = 309		*p*-value	Mothers *N* = 252			Fathers *N* = 57		*p*-value
	T1 (2019)	T2 (2021)		T1 (2019)	T2 (2021)		T1 (2019)	T2 (2021)	
Average pain score^a^	1.57 (1.79)	1.8 (1.9)	0.012*	1.74 (1.9)	2.0 (1.9)	0.040*	0.9 (1.1)	1.2 (1.49)	0.002*
Pain interference, activity^b^	1.5 (2.0)	1.5 (1.8)	0.505	1.6 (2.09)	1.6 (2.2)	0.401	1.0 (1.4)	0.7 (1.1)	0.588
Pain interference, emotions^b^	1.6 (1.9)	1.8 (2.2)	0.007*	1.7 (2.0)	2.0 (2.2)	0.018*	1.1 (1.4)	1.1 (1.4)	0.165
Pain duration			0.014*			0.025*			0.548
No pain	122 (40%)	94 (30%)		94 (37%)	71 (28%)		28 (41%)	23 (40%)	
≤ 3 months	50 (16%)	60 (19%)		41 (16%)	47 (19%)		9 (16%)	13 (23%)	
> 3 months	137 (44%)	155 (50%)		117 (47%)	134 (53%)		20 (35%)	21 (37%)	
Pain analgesics in the past 4 weeks			0.175			0.556			0.115
Yes	83 (60%)	171 (55%)		154 (61%)	148 (59%)		31 (54%)	23 (40%)	
No	124 (40%)	138 (45%)		98 (39%)	104 (41%)		26 (46%)	34 (60%)	
Frequency of pain analgesics in the past 4 weeks			0.805						0.846
Daily	17 (9%)	23 (14%)		14 (9%)	18 (12%)		3 (10%)	5 (22%)	
Every week, but not daily	35 (19%)	34 (20%)		31 (20%)	32 (22%)		4 (13%)	2 (8%)	
Less often than every week	132 (71%)	112 (65%)		108 (70%)	96 (65%)		24 (77%)	16 (70%)	
No intake	1 (0.5%)	2 (1%)		1 (1%)	2 (1%)		-	-	
Family pain			0.317			0.267			0.446
Yes	135 (44%)	148 (48%)		117 (46%)	115 (45%)		18 (32%)	13 (23%)	
No	138 (45%)	128 (41%)		107 (43%)	115 (46%)		31 (54%)	33 (58%)	
Don’t know	36 (11%)	33 (11%)		28 (11%)	22 (9%)		8 (14%)	11 (19%)	
Chronic illness			0.176			0.162			1.000
Yes	76 (25%)	75 (24%)		61 (24%)	59 (24%)		15 (26%)	16 (28%)	
No	230 (74%)	226 (73%)		188 (75%)	185 (73%)		42 (74%)	41 (72%)	
Don’t know	3 (1%)	8 (2%)		3 (1%)	8 (3%)				

Categorical data are presented as number (%) and continuous variables as mean (SD). McNemar tests were used to compare differences in categorical variables and paired sample *t* tests for continuous data

^a^Range: 0–10, where 10 indicates pain as bad as can be imagined

^b^Range 0–10, where 10 indicates complete interference of pain

^*^p ≤ 0.05

### Characteristics of the sample

In total, 309 parents participated in this longitudinal study. When comparing non-responders at T2 with responders, we found that responders had a higher level of education (43% vs 57%), and the proportion of mothers was also higher (73% vs 82%) (see supplementary Table 1). Table 1 shows the descriptive characteristics for all the included parents, stratified by gender, and comparisons between T1 with T2. At T1, most of the responders (81%) were married or cohabitating, had a university education of four years or more (57%), worked full-time (75%) or part-time (17%) and had a household income (53%) of more than NOK1 million. The same patterns were seen at T2 for all the parents and for mothers and fathers separately. We identified no statistically significant changes in sociodemographic variables between T1 to T2 (see Table 1).

When comparing pain-related variables between non-responders and responders at T2, non-responders scored significantly higher on pain interference with activity, 1.69 (95% CI [1.31; 2.07]) vs 1.48 (95% CI [1.19; 1.76]), and pain interference with emotion, 1.82 (95% CI [1.47; 2.18]) vs 1.65 (95% CI [1.37; 1.91], at T1. However, the percentage of parents with pain for a duration of 3 months was significantly higher among the responders (36% vs 44%) (see supplementary Table 2). Description of pain, pain-interference and use of pain medication among the responders is presented in Table 2. From T1 to T2, the average pain score increased, 1.6 (95% CI [1.4; 1.8]) vs 1.8 (95% CI [1.6; 2.0]), the pain interference with emotion score increased, 1.6 (95% CI [1.3; 1.9]) vs 1.8 (95% CI [1.5; 2.1]), and a large proportion reported pain duration of more than 3 months (44% vs 50%, *p* = 0.014). This pattern seemed to be especially prominent among mothers, but the difference in change scores (delta score) between the genders was only statistically significant for the average pain score.

### Psychological factors and health-related quality of life

When comparing psychological and HRQOL data at baseline between non-responders and responders at T2, the only statistically significant difference was revealed for the RAND-36 sub-domain physical function (92.5 (95% CI [90.8; 94.3]) vs 94.1 (95% CI [93.7; 95.5]). For details, see Supplementary Table 3. For the responders, the parents’ scores for psychological factors and HRQOL are listed in Table 3. There were few statistically significant changes between T1 and T2. Compared with T1, at T2, the parents were significantly lonelier, 12.8 (4.2) vs. 13.7 (95% CI[13.2; 14.2]), reported significantly lower RAND-36 scores for the two sub-domains, mental health score, 81 (95% CI [80; 82]) vs 80 (95% CI [78; 81]), and emotional role function, 89 (95% CI [86; 92]) vs 82 (95% CI [79; 86]), and the sum score RAND-36 MCS, 52.2 (95% CI [51.3; 53.2]) vs. 50.9 (95% CI [49.8; 52.0]). The changes in emotional role function were significantly more prominent among women than men. No statistically significant changes in the RAND-36 PCS were seen.

**Table 3:** Descriptive characteristics of HRQOL, self-efficacy, self-esteem, loneliness and stress (*N* = 309) and differences between women (*N* = 252) and men (*N* = 57)

	All T1 (2019) *N* = 309	All T2 (2021)	*p*-value	Mothers T1 (2019) *N* = 252	T2 (2021)		Fathers T1 (2019) *N* = 57	T2 (2021)	*p*-value
HRQOL									
RAND-36 PCS^a^	52 (9)	52 (8)	0.793	51.4 (9.1)	51.6 (9.1)	0.690	54.8 (5.3)	54.6 (5.0)	0.592
RAND-36 MCS^a^	52.2 (8.2)	50.9 (9.7)	0.008*	51.8 (8.5)	50.3 (10.2)	0.012*	54.2 (6.5)	53.9 (6.6)	0.408
RAND-36 eight domains									
Bodily pain	79 (22)	78 (23)	0.435	77.1 (23.4)	76.6 (24.3)	0.686	87.4 (14.9)	85.0 (14.8)	0.221
General health	77 (20)	76 (20)	0.102	75.8 (20.9)	74.9 (20.7)	0.265	83.3 (12.8)	80.9 (14.2)	0.171
Physical function	94 (13)	93 (14)	0.149	92.4 (13.5)	92.5 (13.7)	0.131	97.2 (6.1)	97.3 (5.5)	0.332
Physical role function	85 (32)	84 (31)	0.652	83.1 (33.7)	81.5 (33.4)	0.502	92.5 (22.1)	94.7 (18.7)	0.849
Mental health	81 (12)	80 (14)	0.025*	80.4 (12.6)	79.0 (14.8)	0.093	84.7 (9.5)	82.0 (9.7)	0.245
Vitality	64 (21)	63 (20)	0.455	62.0 (20.7)	61.6 (21.3)	0.718	71.2 (14.9)	69.1 (15.2)	0.242
Social function	87 (19)	87 (20)		85.6 (20.7)	85.2 (21.4)	0.716	94.7 (9.3)	93.4 (11.6)	0.272
Emotional role function	89 (27)	83 (34)	0.001*	89.0 (27.2)	80.0 (36.2)	< 0.001*	91.2 (25.6)	94.2 (20.0)	0.403
Psychological factors									
General self-efficacy ^b^	3.3 (0.4)	3.29 (0.6)	0.219	3.3 (0.4)	3.3 (0.4)	0.153	3.4 (0.5)	3.4 (0.4)	0.869
Loneliness ^c^	12.8 (4.2)	13.7 (4.3)	< 0.001*	12.9 (4.4)	17.7 (4.3)	< 0.001*	12.5 (4.2)	13.7 (4.2)	< 0.001*
Stress ^d^	0.28 (0.16)	0.28 (0.16)	0.613	0.29 (0.16)	0.28 (0.16)	0.602	0.24 (0.14)	0.24 (0.49)	0.962
Self-esteem ^e^	3.35 (0.58)	3.32 (0.57)	0.180	3.3 (0.5)	3.5 (0.6)	0.224	3.5 (0.5)	3.46 (0.49)	0.578

Paired samples *t-* tests were used to compare T1 with T2 for all participants and for mothers and fathers separately

^a^The score for the SF-36 ranges from 0 to 100, where 100 indicates high HRQOL. PCS, physical component summary; MCS, mental component summary

^*^p ≤ 0.05

### Baseline characteristics predicting two-year changes in HRQOL

Table 4 shows the adjusted associations between gender, sociodemographic factors, pain, self-esteem, self-efficacy, loneliness, stress (predictors) and two-years changes in HRQOL (delta RAND-36 PCS and MCS). Positive change in MCS was predicted by baseline characteristics of working part time, B = 5.22 (95% CI [1.05;9.38)]) and older age, B = 0.24, (95%CI [-001, 0.42]), and a negative change was predicted by stress, B = -17.39, (95%CI [-27.42, -7.51]). We identified no statistically significant baseline predictive factors of changes in PCS.

**Table 4:** Adjusted associations between gender, demographic variables, psychosocial variables, pain and changes in HRQOL (delta RAND PCS or MCS) examined by linear regression analyses, *N* = 309

		Delta RAND-36 PCS			Delta RAND-36 MCS	
	B	(CI)	*p*-value	B	(CI)	*p*-value
Gender (Ref = father)	-0.17	-2.03, 1.70	0.861	-1.28	-3.68, 1.17	0.297
Age	-0.08	-0.26, 0.10	0.379	0.24	0.01, 0.47	0.038*
Region (ref = Oslo/Viken)	-0.19	-1.81, 1.42	0.818	0.65	-1.42, 2.74	0.537
Living conditions						
Married/cohabitating	ref			ref		
Single/divorced, widow/widower	1.52	-0.81, 3.86	0.201	-0.67	-3.69, 2.34	0.660
Education						
Less than 13 years of education	-0.95	-3.14, 1.24	0.395	0.06	-2.76, 2.88	0.967
University less than 4 years	-0.15	-1.87, 1.58	0.869	-0.49	-2.73, 1.70	0.666
University 4 years or more	Ref			Ref		
Employment status (ref = Not paid work)						
Full-time	1.53	-1.82, 4.52	0.401	3.64	-0.21, 7.48	0.064
Part-time	0.70	**-**2.68, 4.07	0.686	5.22	1.05, 9.38	0.014*
Absence from work (ref = 0 days)						
1–4 days	-0.83	-2.58, 0.92	0.352	-0.91	-3.17, 1.34	0.426
5–7 days	-2.22	-6.42, 2.01	0.303	-3.85	-9.33, 1.64	0.169
8–10 days	1.37	-5.71, 8.44	0.704	-8.14	-17.27, 1.00	0.081
Household income (NOK)						
Less than 250,000	ref			ref		
250,000–450,000	2.43	-10.11, 14.97	0.703	4.28	-12.03, 20.88	0.606
451,000–750,000	4.36	-8.26, 16.98	0.497	1.46	-14.91, 17.84	0.861
751,000–1,000,000	4.54	-8.23, 17.32	0.484	3.33	-13.26, 19.92	0.693
More than 1,000,000	5.29	-7.62, 18.19	0.421	2.49	-14.27, 19.26	0.770
Self-efficacy ^a^	-0.45	-2.36, 14.7	0.646	1.18	-1.31, 3.67	0.351
Self-esteem ^b^	0.18	-0.27, 0.64	0.430	0.10	-0.49, 0.69	0.738
Pain (ref = none)						
Less than 3 months	-0.36	-2.50, 1.78	0.740	1.62	-1.12, 4.36	0.246
More than 3 months	-1.23	-2.99, 0.52	0.168	-0.86	-2.99, 1.28	0.430
Loneliness ^c^	0.11	-0.16, 0.32	0.334	-0.08	-0.36, 0.20	0.594
Stress ^d^	-4.28	-10.81, 2.24	0.197	-17.39	-27.42, -7.35	< 0.001*
RAND-36 PCS at baseline ^e^	-0.367	-4.48, -0.26	< 0.001			
RAND-36 MCS at baseline ^e^				-0.76	-0.794, 0.58	< 0.001*
R^2^ adj	12.7%			19.2%		

^a^Self-efficacy: range 1–4, where higher values indicate higher levels of self-efficacy

^b^Self-esteem: range 1–4, where higher values indicate higher levels of self-esteem

^c^Loneliness: range 8–32, where higher values indicate higher levels of loneliness

^d^Stress: range 0–1, where higher values indicate higher levels of stress

^e^The score for the SF-36 ranges from 0 to 100, where 100 indicates a high HRQOL. *PCS* Physical component summary, *MCS* Mental component summary

^*^p ≤ 0.05

## Discussion

During the two-year period, we identified some changes in sociodemographic and psychological factors, pain and HRQOL in parents of adolescents. The parents’ average pain score was rather low, but it increased during the study period; the parents were lonelier and more reported a decrease in mental HRQOL. The baseline predictor of decreased mental HRQOL was stress, while paid work and older age predicted an increase in mental HRQOL. Our data did not reveal any statistically significant predictive factors associated with changes in physical HRQOL.

Our results showed rather small changes in sociodemographic and psychological factors, pain and HRQOL between 2019 and 2021, although most were of limited clinical significance. We assumed that fear of infection and serious illness, in combination with imposed home offices, closed schools and leisure activities for young people and periods with closed shops and restaurants, might increase stress and reduce HRQOL in parents. However, the Norwegian state’s strategy and ability to secure jobs and income for most people had been good, which may have had an impact on the population’s HRQOL during the pandemic [48]. Security in financial matters and the fact that the pandemic situation did not affect their family’s social status may have been factors contributing to a relatively stable HRQOL in the parents in our study. Another Norwegian study has pointed out that health-related risks and work–life balance played predominant roles in predicting life satisfaction before and during the pandemic [49]. And the same study showed that during the pandemic, people with poor health experienced worsened work circumstances, compared with people from a healthy population [49]. The parents who participated in this study were characterised as having high socio-economic status and higher education, which may have been buffers against stress caused by external conditions such as the pandemic.

The average pain score increased significantly for mothers and fathers during the study period. Still, the increase was small, the level of pain remained rather low at both time points and there was no increase in the use of pain analgesics. The average pain scores in the sample of mothers and fathers at T2 were 2.0 (mothers) or below (fathers) on a 0–10 pain scale. Intensity ratings for pain, or levels of pain, are often described as mild, moderate and severe, and much research has been done to establish the cut points for mild, moderate and severe in different pain populations. Such cut points are used to guide pain treatment in clinical practice. Palos et al. [50] studied how ‘healthy’ adults rated pain severity cut points and found them to be 1 to 4 for mild pain, 5 to 6 for moderate pain and 7 to 10 for severe pain—very similar to the cut points in various clinical samples. Following this, an average pain score between 0 and 2 would mean mild pain for both mothers and fathers in our study. Even though a negative relationship between pain and HRQOL has been described in several studies [51], the findings of our study showed no significant associations between average pain and changes in HRQOL over time. These results may be explained by the relatively low pain intensity scores for both mothers and fathers.

Our results further show that the parents were lonelier at T2 than they were at T1. Loneliness is a significant public health issue and associated with a wide range of health outcomes, such as mental health problems, substance use and physical health conditions [52]. Our findings correspond with earlier studies performed during the pandemic that have demonstrated an increase in prevalence of loneliness and social disconnection over that time [53]. However, even though a negative change was seen among parents in our study, the association with HRQOL was not significant. One explanation for this may again be the high socio-economic status in the participating parents. Loneliness is a key risk factor likely to have an impact on mental health and HRQOL, particularly on those who are vulnerable due to pre-existing health problems and/or low socioeconomic status [54].

Stress was a negative predictor of changes in the parents’ mental HRQOL. Previous studies have shown that parental subjective mental health status correlates significantly with parent–child relationships and financial resources [55]. Work stress and imbalance between work and family/personal life have been found to increase mental health problems in the working population [56]. Moreover, studies have shown that loneliness and mental health problems in adolescents have increased during covid [57–60], and many parents may have been concerned about their children’s health during the pandemic, which may be a stress element that can affect their HRQOL. Stress over time and maladapting to stressful environments may therefore have serious consequences [61].

As seen in our cross-sectional baseline study of the cohort [27], a strong work affiliation is also important for parental HRQOL over time. It may reflect a feeling of commitment and desire to contribute to society, along with the self-respect that paid work brings. Paid work implies income, sustenance and safety [62], which might have been especially important during the COVID-19 pandemic. Being in paid work has been identified as important for HRQOL in the general population, whereas absence from work, possibly because of health problems, is considered to have the opposite effect [4, 5, 62, 63].

Consider our findings in the light of the revised Wilson and Cleary model for health-related quality of life [26], which suggest relationships between symptoms, functioning, characteristics of the individual and the environment and HRQOL, we identified some associations. For example, characteristic with the individual like a strong work affiliation and old age were associated with positive change in mental HRQOL, while stress, which might be considered a symptom, was negatively associated. No associations between pain and changes in HRQOL might have been buffered by resilience factors within the person or the environment, like self-efficacy and self-esteem.

### Strengths and limitations

The main strengths of this study are its longitudinal design and high number of potential included predictive factors, all assessed using validated instruments. On the other hand, a follow-up period of two years might be considered to be a too short time to reveal changes and an additional time of measures might have brought other results. The overall response rate of 55% may be considered a limitation, as attrition within longitudinal studies may deteriorate the generalisability of findings [64]. Furthermore, we identified some differences in the baseline scores for demographic factors and pain between responders and non-responders.‘As listed in Supplementary table 2, there were statistically significant differences between responders and non-responders. More non-responders had lower education, lower physical function, reporting that their pain influenced them both regarding activities and emotions, at the same time fewer reported long-lasting pain which might limit the generalizability of our results. However, this possible selection bias is very common in population-based studies, as for example in a very large Norwegian, population based MoBa study [65]. The differences might indicate an even a higher percentage of parents with higher socioeconomic status responding to both time points (see supplementary tables). It is important to note that most participants were mothers. Further, the majority were married/cohabitating, employed, well-educated and with a high household income, indicating high socioeconomic status, especially at T2. Thus, our results may not be representative of the entire population of Norwegian parents, which should be considered when interpreting our results. We did not include any characteristics of the parents’ adolescent, which might have been considered as simplifying of their situation, and more characteristics might have brought a more nuanced finding, and which in retrospective are considered a weakness. All the self-reported measures are reliable and valid, psychometric testing performed in previous international and national studies[35, 36, 39, 40, 45]. However, looking closer into the validation papers of the questionnaires, few of them have reported a CFA [31, 46, 66], which must be considered a weakness. For the validated measures [35, 36, 39, 40, 45] used to assess the predictors of HRQOL, we chose to use them as initially designed with continuous variables remaining continuous and not using previous suggested cut-of values.

## Conclusion

To conclude, this study describes how sociodemographic and psychological factors, pain and HRQOL in parents changed over a two-year period during the COVID-19 pandemic. Interestingly, even if we identified more pain and loneliness, especially in mothers, the association with HRQOL was of limited clinical significance and influenced HRQOL to a limited extent. Special attention should be paid to vulnerable families with children or parents with pre-existing health problems. This is an area that warrants further investigation.

## Supplementary Information

**Additional file 1**: **Table 1**. Baseline characteristics of the non-responders (*n*=252) versus responders (*n*=309) at 2-year follow-up. **Table 2**. Description of pain in of the non-responders (*n*=252) versus responders (*n*=309) at 2-year follow-up. **Table 3**. Descriptive characteristics of HRQOL, self-efficacy, self-esteem, loneliness and stress of the non-responders (*n*=252) versus responders (*n*=309) at 2-year follow-up.

## Data Availability

The datasets used and/or analyzed during the current study are not publicly available due to General Data Protection Regulation laws but are available from the corresponding author on reasonable request and with permission from the Norwegian Centre for Research Data.

## Abbreviations

BPI: Brief Pain Inventory
GSE: General self-efficacy scale
HRQOL: Health-related quality of life
LPQ: Lübeck Pain-Screening Questionnaire
OTC analgesics: Over-the-counter analgesics
PSQ: Perceived Stress Questionnaire
QOL: Quality of life
RSES: Rosenberg Self-Esteem scale
SES: Socioeconomic status
SUS: Pain youth and self-medication study
ULS: UCLA Loneliness Scale
WHO: World Health Organization
