# Supplementary material for: Health-related quality of life in parents of adolescents one year into the COVID-19 pandemic: a two-year longitudinal study
Source: Health Qual Life Outcomes. 2022 Dec 1;20:158. doi: 10.1186/s12955-022-02069-8 (PMC9713202; doi:10.1186/s12955-022-02069-8)
Supplement: Supplementary file 1 — Additional file 1: Table 1. Baseline characteristics of the non-responders (n=252) versus responders (n=309) at 2-year follow-up. Table 2. Description of pain in of the non-responders (n=252) versus responders (n=309) at 2-year follow-up. Table 3. Descriptive characteristics of HRQOL, self-efficacy, self-esteem, loneliness and stress of the non-responders (n=252) versus responders (n=309) at 2-year follow-up. [file 12955_2022_2069_MOESM1_ESM.docx]

**Table 1** Baseline characteristics of the non-responders (n=252) versus responders (n=309) at 2-year follow-up

| Demographic | Non-responders N=252 | Responders N=309 | p-value |
| --- | --- | --- | --- |
| Mothers | 184 (73%) | 252 (82%) | 0.016* |
| Fathers | 68 (27%) | 57 (18%) |  |
| Age, years mean (SD) | 45.04 (5.26) | 45.54 (4.36) | 0.019* |
| *Living condition* |  |  | 0.382 |
| Married/cohabitating | 208 (83%) | 249 (81%) |  |
| Single | 16 (6%) | 17(5%) |  |
| Divorced or separated | 24 (9%) | 31 (13%) |  |
| Widowed | 4 (2%) | 2 (1%) |  |
| *Education* |  |  | <0.001* |
| Compulsory education, post-compulsory or certificate of apprenticeship | 79 (31%) | 58 (19%) |  |
| University <4 years | 66 (26%) | 75 (34%) |  |
| University ≥4 years | 107 (43%) | 176 (57%) |  |
| *Employment status* |  |  | 0.444 |
| Full time | 181 (72%) | 233 (75% |  |
| Part-time | 53 (21%) | 52 (17%) |  |
| Not working | 18 (7%) | 24 (8%) |  |
| *Absence from work last 3 months* |  |  | 0.990 |
| None | 169 (67%) | 203 (66%) |  |
| 1–4 days | 55 (22%) | 6 (22%) |  |
| 5–7 days | 8 (3%) | 9 (1%) |  |
| 8–10 days | 2 (1%) | 3 (1%) |  |
| More than 10 days | 18 (7%) | 25 (8%) |  |
| *Household income (NOK)* |  |  | 0.376 |
| <250,000 | 4 (2%) | 1 |  |
| 250,000–450,000 | 23 (9%) | 20 (7%) |  |
| 451,000–750,000 | 44 (17%) | 52 (17%) |  |
| 751,000–1,000,000 | 57 (23%) | 72 (23%) |  |
| >1,000,000 | 124 (49%) | 164 (53%) |  |

Categorical data are presented as number (%) and continuous variables as mean (SD).

Chi-square-tests were used to compare differences in categorical variables and independent sample *t* tests for continuous data

* P ≤ 0.05

**Table 2** Description of pain in of the non-responders (n=252) versus responders (n=309) at

2-year follow-up

|  | Non-responders N=252 | Responders N=309 | p-values |
| --- | --- | --- | --- |
| Average pain score ^a^ | 1.18 (1.85) | 1.14 (1.80) | 0.708 |
| Pain interference, activity ^b^ | 1.69 (2.36) | 1.48 (1.98) | 0.022* |
| Pain interference, emotions ^b^ | 1.82 (2.26) | 1.65 (1.90) | 0.024* |
| Pain duration |  |  |  |
| No pain | 101 (40%) | 122 (40%) | 0.040* |
| ≤3 months | 60 (24%) | 50 (16%) |  |
| >3 months | 91 (36%) | 137 (44%) |  |
| Pain analgesics in the past 4 weeks |  |  | 0.349 |
| Yes | 141 (56%) | 185 (60%) |  |
| No | 111 (44%) | 124 (40%) |  |
| Frequency of pain analgesics in the past 4 weeks |  |  | 0.070 |
| Daily | 9 (6%) | 17 (9%) |  |
| Every week, but not daily | 43 (31%) | 35 (19%) |  |
| Less often than every week | 87 (62%) | 13 (71%) |  |
| No intake | 2 (1%) | 1 (1%) |  |
| Family pain |  |  | 0.224 |
| Yes | 95 (38%) | 135 (43%) |  |
| No | 131 (52%) | 138 (45%) |  |
| Don’t know | 26 (10%) | 36 (12%) |  |
| Chronic illness |  |  | 0.167 |
| Yes | 52 (21%) | 76 (25%) |  |
| No | 193 (76%) | 230 (74%) |  |
| Don’t know | 7 (3%) | 3 81%) |  |

Categorical data are presented as number (%) and continuous variables as mean (SD).

Chi-square-tests were used to compare differences in categorical variables and independent sample *t* tests for continuous data.

^a^ Range: 0–10, where 10 indicates pain as bad as can be imagined.

^b^ Range 0–10, where 10 indicates complete interference of pain.* P ≤ 0.05

**Table 3:** Descriptive characteristics of HRQOL, self-efficacy, self-esteem, loneliness and stress of the non-responders (n=252) versus responders (n=309) at 2-year follow-up

|  | Non-responders N=252 | Responders N=309 | p-values |
| --- | --- | --- | --- |
| HRQOL |  |  |  |
| RAND-36 PCS^a^ | 51.3 (9.2) | 52.0 (8.6) | 0.494 |
| RAND-36 MCS^a^ | 52.2 (8.2) | 52.2 (8.2) | 0.878 |
| RAND-36 eight domains |  |  |  |
| Bodily pain | 77.8 (24.3) | 79.1 (22.4) | 0.443 |
| General health | 76.1 (19.0) | 77.2 (19.9) | 0.495 |
| Physical function | 92.5 (14.1) | 94.1 (12.6) | 0.043* |
| Physical role function | 82.8 (34.6) | 84.9 (32.0) | 0.189 |
| Mental health | 80.9 (13.2) | 81.2 (12.2) | 0.564 |
| Vitality | 64.0 (20.5) | 63.7 (20.1) | 0.947 |
| Social function | 86.5 (20.6) | 87.3 (19.4) | 0.246 |
| Emotional role function | 89.0 (28.4) | 89.4 (26.9) | 0.654 |
| Psychological factors |  |  |  |
| General self-efficacy^b^ | 3.39 (0.39) | 3.31 (0.42) | 0.990 |
| Loneliness ^c^ | 12.6 (4.2) | 12.83 (4.4) | 0.905 |
| Stress ^d^ | 0.27 (0.15) | 0.28 (0.16) | 0.538 |
| Self-esteem ^e^ | 3.33 (0.57) | 3.35 (0.53) | 0.252 |

Independent samples *t-* tests were used to compare non-responders and responders.

^a^ The score for the SF-36 ranges from 0 to 100, where 100 indicates a high HRQOL. PCS, physical component summary; MCS, mental component summary.

^b^Self-efficacy: range 1–4, where higher values indicate higher levels of self-efficacy

^c^ Self-esteem: range 1–4, where higher values indicate higher levels of self-esteem.

^d^ Loneliness: range 8–32, where higher values indicate higher levels of loneliness.

^e^ Stress: range 0–1, where higher values indicate higher levels of stress.

* P ≤ 0.05
